# Integrated Analyses Reveal Potential Functional N6-Methyladenosine-Related Long Noncoding RNAs in Adrenocortical Adenocarcinoma

**DOI:** 10.3389/fcell.2022.851748

**Published:** 2022-05-20

**Authors:** Yafei Ding, Tao Wang, Yuankang Feng, Xiaohui Ding, Xiang Li, Zhenlin Huang, Zhankui Jia, Jun Wang, Jinjian Yang

**Affiliations:** Department of Urology, the First Affiliated Hospital of Zhengzhou University, Zhengzhou University, Zhengzhou, China

**Keywords:** adrenocortical carcinoma (ACC), LncRNA, long noncoding RNA, N6-methyladenosine (m 6 A), risk model, LASSO

## Abstract

**Background:** Adrenocortical adenocarcinoma (ACC) is known to be a relatively uncommon malignant tumor of the adrenal gland with patients having a poor prognosis. At present, few reports are available concerning the m6A modifications of lncRNAs as well as their clinical and immunological significance in the occurrence and progression of ACC.

**Materials and Methods**: In the present research, 21 m6A-related genes were analyzed. Both multivariate and univariate Cox regression analyses were conducted to examine the prognostic m6A-related lncRNAs. A sum of 165 m6A-related lncRNAs was obtained from The Cancer Genome Atlas (TCGA) dataset. Based on the expressions of m6A-related lncRNAs, all ACC patients were classified into distinct subgroups using the consistent clustering method. Finally, m6A-related lncRNAs that were shown to have prognostic value were utilized to develop an m6A-related lncRNA risk model, which may be employed in the prediction of prognosis and survival.

**Results:** Using TCGA data set, 26 m6A-associated lncRNAs having putative prognostic values were identified according to their expression levels, TCGA-AAC patients were classified into two clusters with the aid of consistent clustering analysis. The correlation between the two clusters was low, in which cluster1 consisted of 42% of all ACC patients. The survival analysis showed that cluster1 was associated with an unfavorable prognosis relative to cluster2. A risk model was constructed incorporating 26 m6A-associated lncRNAs that were correlated with patient prognosis. The model was subsequently validated by univariate and multivariate Cox, receiver operating characteristic (ROC) curve, and survival analyses. We also observed that the m6A-related risk model performed well in anticipating prognoses and survival status of patients with AAC. The overall survival (OS) of the high-risk cohort, as predicted by the model, was lower as opposed to that of the low-risk cohort.

**Conclusion:** In the present research, we developed a risk model consisting of 4 m6A-related long-noncoding RNAs (lncRNAs), which can exert independent predictive values in patients with ACC. Our findings demonstrated that these 4 m6A-related lncRNAs perform integral functions in the tumor immune microenvironment, and also revealed the possibility of using these lncRNAs to guide the development of prognostic classifications and therapy approaches for ACC patients.

## Introduction

Adrenocortical carcinoma (ACC) is an uncommon malignant tumor. The annual incidence rate is approximately 0.7–2.1 per million individuals (Veytsman et al., 2009). Its clinical manifestations are strongly heterogeneous, and these patients have relatively poor prognoses (Else et al., 2014; Altieri et al., 2020). They are prone to early distant metastasis, resulting in unsatisfactory clinical outcomes. The 5-years survival rate is as low as 15–44% (Fassnacht et al., 2018), and the prognostic factors remain largely unclear. At present, the main treatment method of ACC is radical surgery. There is a lack of effective adjuvant treatment and the incidence of postoperative recurrence and distant metastases are high, resulting in difficulty for follow-up clinical treatment. Although research on ACC has deepened in recent years, still, there is much room for improvement in the diagnoses, treatment, and prognoses of patients with ACC(Fassnacht et al., 2020). In view of this, the identification of highly specific biological markers for the diagnosing and treating of ACC is critical.

N6-methyladenosine (m6A) methylation is a kind of significant epigenetic alteration in RNAs such as lncRNAs, which was first discovered in 1974 (Roundtree et al., 2017; He et al., 2019). In recent years, m6A methylation has become a research hotspot in the field of life sciences. It is common for mammalian cells to reverse the methylation of N6-methyladenosine on a dynamic basis; its epigenetic regulatory mechanisms are similar to those of DNA and histone modifications. This RNA chemical marker is produced by the proteins belonging to m6A “writers” (METTL14, WTAP, METTL16, etc.). These modifications may be reversed by m6A “erasers” (demethylase, ALKBH5, FTO). In addition, “readers” (YTHDC, YTHDF1, etc.) can recognize the m6A-containing mRNAs and thus modulate the expression of the downstream genes (Wang et al., 2020). The methylation of m6A-RNA participates directly in all the RNA life cycle phases, from processing, nuclear output, translational regulation, and finally, degradation, thereby indicating its roles in RNA metabolism. Recent studies have shown that m6A modification is a complex regulatory network in several cancer types, and is strongly associated with tumorigenesis, progression, metastasis, as well as the invasion of tumors (Huang et al., 2020). Emerging evidence confirms that m6A-related genes are significantly abnormally regulated in ACC(Jin et al., 2021; Shen et al., 2021), thus influencing the patients’ prognoses. Nonetheless, the regulatory mechanisms are unclear.

Long non-coding RNA (lncRNAs) are RNAs with lengths exceeding 200 nt; these RNAs are involved in transcriptional regulation whereas they have no obvious protein-coding functions (Quinn and Chang, 2016). The imbalance in lncRNAs might perform an integral function in tumorigenesis of all tumor types, including prostate, gastric, and non-small cell lung cancers (Hua et al., 2019; Chen et al., 2020; Li et al., 2020; Wei et al., 2020; Wen et al., 2020). Moreover, recent research reports show that lncRNAs might be involved in modulating the upstream and downstream expressions of m6A-related genes (Dai et al., 2020). Both m6A-related genes and lncRNAs are excellent diagnostic and prognostic indicators. A growing body of research suggests that m6A-related mRNAs and lncRNAs may function as viable possible targets for predicting prognoses in a variety of malignancies (Ni et al., 2019). Therefore, systematic identification of the major m6A-related lncRNAs and m6A regulators in ACC using the high-throughput data, examination of the prospective modulatory mechanisms correlated with lncRNA and m6A regulators, and probing into the functions performed by the m6A-related lncRNAs in the tumorigenesis as well as the progression of ACC, is crucial.

In the present research, we employed data obtained from The Cancer Genome Atlas (TCGA) and the Genotype-Tissue Expression (GTEx) project for the purpose of creating an m6A-related predictive lncRNA model for ACC. We validated the predictive power of the m6A-related lncRNA model. Moreover, the functions of lncRNAs incorporated in the model were validated in two ACC cell lines (NCI-H295R and SW-13). Overall, our findings suggest that a robust m6A-related prognostic signature comprised of lncRNAs can be used to anticipate OS in patients with ACC. These findings might provide suggestions for future mechanistic research focusing on the specific functions of m6A modified-lncRNAs.

## Materials and Methods

### Data Acquisition

The sequencing data as well as the matching clinical data for all patients from The Cancer Genome Atlas (TCGA) and the GTEx project were obtained from the UCSC Xena site (https://xena.ucsc.edu/, update to 2021.10.31). Patients who did not have documented survival data were not included in the present research. The expression levels of genes were normalized as log2 (1 + FPKM value).

### Differentially Expressed m6A-Related Regulators

On the basis of prior publications, the expression matrixes for 21 m6A-related genes were obtained from the gene expression matrix and subjected to subsequent analyses. Before performing differential expression analyses, the genes exhibiting low abundances were eliminated from consideration. The differential expression analysis was carried out by means of the limma (version: 3.48.3) program in the R software. Calculation of the differences was performed with the aid of log2 fold change (FC) and Student *t*-test. We then determined that genes having an adjusted *p*-value of less than 0.05 and a log2 (FC) greater than 0.5 represented a significant differential expression. The Pearson technique in R was used for the purpose of conducting the correlation analyses.

### Annotation of lncRNAs

The Gencode website (https://www.gencodegenes.org/) was utilized to acquire the lncRNA annotation file of genome reference alliance human construction 38 (GRCh38), which was then used to annotate the lncRNAs obtained from the TCGA dataset. A sum of 14,247 lncRNAs was identified according to the integrated ID of these genes.

### Consensus Clustering

The ConsensusClusterPlus (version: 1.56.0) module in R was utilized to identify the subtypes of cancer with the aid of the consensus clustering algorithm. By examining the delta area plot, we successfully calculated the optimal clustering number.

### Comparison of the Immune Status

The TCGA-ACC samples were analyzed using the CIBERSORT method (https://cibersort.stanford.edu/). In this way, we determined the proportion of 22 distinct immune cell subtypes present. We obtained the proportions of the 64 different immune cell types by uploading the gene expression matrix to the website in a direct manner and calculating the results. The Kruskal–Wallis test was employed for the purpose of performing comparisons of the differences between the patients with high- and low-risk scores or among different subgroups. A univariate Cox regression analysis was performed on the significantly distinct cell types found in low- and low-m6A-LPS tumors so as to determine their relationship with OS.

### Computation of Prognostic Risk Scores and Clinicopathological Correlations

We used the createDataPartition function in the R package Caret to divide the dataset into a training set and a validation set. The parameter was set to *p* = 0.5, i.e., half of the samples were assigned to the training and validation sets, respectively. The least absolute shrinkage and selection operator (LASSO) Cox regression analysis was carried out using R utilizing the glmnet package (the 10-fold cross-validation was used for the purpose of estimating the penalty parameter). For patients with ACC, we established an m6A-related lncRNA prognostic signature (m6A-LPS) consisting of four m6A-related lncRNAs. The following equation was used to determine the risk scores:

$$Risk\;score=\overset{n}{\underset{k=1}{\sum }}coef_{k}*x_{k}$$

where Coef_k_ denotes the coefficients and X_k_ denotes the FPKM value for each m6A-related lncRNA.

To determine the predictive validity regarding the risk model, the survival analysis and receiver operating characteristic (ROC) curve analysis were conducted. The prognostic ability of the OS predictive model was assessed by the ROC curve (timeROC package in R) and the value of the area under the curve (AUC). The AUC value denotes the area under the ROC curve, we used the R package pROC to calculate the AUC 95% CI values. Both univariate and multivariate Cox regression analyses were employed to examine the independent prognostic capacity of risk scores. The above analyses were conducted in R (version:4.1.2). Additionally, we utilized the GSEA software to examine tumor characteristics that were highly prevalent in the high-risk cohort in contrast with those in the low-risk cohort.

### Statistical Analysis

The log-rank test, as well as the Kaplan–Meier curves, were utilized for comparing the OS among subgroups consisting of the low-and high-risk patients, classified according to their median expression levels of m6A-related lncRNAs. For the purpose of comparing risk scores (derived from the m6A-LPS) among cohorts, the Student’s *t*-test was utilized. We conducted multivariate and univariate Cox regression analyses to determine the independent prognostic significance of m6A-LPS for anticipating OS.

### Cell Culture

The American type culture collection (ATCC, USA) supplied the human ACC cell lines, SW-13, and NCI-H295R. These cell lines were incubated at a temperature of 37°C in a water-saturated environment that contained 5% CO_2_ in the RPMI1640 solution (Gibco, United States). Moreover, 10 percent fetal bovine serum (Gibco, United States) was added to the medium.

### Western Blotting

Cultured cells were lysed with RIPA buffer (Solarbio, Beijing, China) supplemented with NaF and protease inhibitor cocktail. The lysates containing equal amounts of protein (25–30 mg/well) were loaded and separated by PAGE Gel Fast Preparation Kit (Epizyme, China). Then the lysates were transferred onto the nitrocellulose membrane. The prepared membrane was blocked with NcmBlot blocking buffer (Epizyme, China) for at least 15 min and then incubated with a specific antibody against E-cadherin (1:50, Abcam), N-cadherin (1:5,000, Abcam) and Vimentin (1:1,000, Abcam) at 4°C overnight. After washing the membranes thrice with TBST for 6 min each, the membranes were incubated with Goat anti-rabbit IgG H&L (IRDye^®^ 800CW) (1: 5,000, Abcam) or Goat anti-Mouse IgG H&L (IRDye^®^ 800CW) (1: 20,000, Abcam).

### Overexpression of Al359643.3

The cDNA of Al359643.3 was cloned into the puromycin-resistant lentiviral vector (pLVX-Puro). The cells were cultured to 50–70% confluence, then they were transfected with jetPRIME^®^ in NCI-H295R and SW-13 cell lines following the manufacturer’s instructions. After 8 h, refresh the Roswell Park Memorial Institute (RPMI) 1,640 medium (Gibco, United States).

### Cell Proliferation Assay

EdU incorporation and CCK8 assays were used to assess cell proliferation. The siRNAs were designed by RiboBio biological Co., Ltd. (https://www.ribobio.com/). The cell lines were cultured and transfected with si-NC, si-RNA1, and si-RNA2 constructs. Once the cells had been transfected, they were transferred into 96-well plates (at a density of 4 × 10^3^ cells/well). Following the incubation of the wells for 24 h, 10 µl of CCK-8 reagent was introduced into each well, followed by incubation of the cells for an additional 2 h at 37°C for specified periods (0, 24, 48, 72, and 96 h). Subsequently, a microplate reader was employed to determine the optical density (OD) measurements at 450 nm. For the EdU incorporation assay, the cells were maintained for 48 h in the culture medium. The cell proliferation was determined on the Cell-Light™ EdU Apollo^®^ 488 platform. When calculating the positive fluorescence area, each data point reflected the area estimated from at least five fields selected at random from three separate tests.

### Colony Formation Assay

Cells that had been subjected to transfection were extracted following trypsinization, enumerated, and seeded at a density of 500 cells per 6-cm dish. After every 3 days, the medium was replaced by a new one. After 10 days, the cells were rinsed two times in 1% PBS, followed by fixing with 3.7 percent methanol, staining with 0.1 percent crystal violet, and enumeration for the purpose of determining the cell count (each colony contained at least 50 cells).

### Transwell Assay

The Transwell assay was carried out on 24-well plates equipped with a membrane filter chamber having an aperture of 8 μm. Matrigel™ (BD Biosciences) was utilized to precoat the top chamber for the purpose of conducting the invasion assay. The cancer cells were plated in the top chamber at a density of 5×10^4^ cells/well in 100 μl FBS-free RPMI1640 culture medium, whereas the bottom chamber was filled with 600 μl RPMI1640 culture medium with 10% FBS. Incubation of the chambers was performed for 24 h at a temperature of 37°C, following which, the cells in the upper chamber were wiped away utilizing cotton swabs. Then, fixing of the cells that had already been attached to the under layers of the membranes was performed using methanol, followed by staining with crystal violet. Ultimately, the images of cells were observed under the microscope.

## Results

### The Expression of N6-Methyladenosine Regulators in ACC Patients

In the present research, since the TCGA dataset did not include tissue samples from the normal adrenal cortex, we obtained datasets from TCGA and GTEx, including 79 ACC and 127 normal tissues (Table 1). First, a total of 21 well-recognized m6A regulators were obtained once the RNA-seq data of ACC patients had been downloaded from the datasets, including the following 8 m6A writers: RBM15B, RBM15, ZC3H13, WTAP VIRMA, METTL16, METTL14, and METTL3; 11 readers: IGFBP1/2/3, LRPPRC, YTHDC1/2, RBMX, YTHDF1/2/3, HNRNPA2B1, FMR1, and HNRNPC, and two erasers: FTO and ALKBH5.

**TABLE 1:** Overview of the clinical information of TCGA and GTEx cohorts.

Characteristics	TCGA (n, %)	GTEx (n, %)
Gender		
Male	30 (37.97%)	75 (59.06%)
Female	49 (62.01%)	52 (40.94%)
Age		
>65	9 (11.39%)	-
≤65	70 (88.61%)	-
Stage		
I	9 (11.39%)	-
II	38 (48.10%)	-
III	17 (21.52%)	-
IV	15 (18.99%)	-
T stage		
T1	10 (12.66%)	-
T2	43 (54.43%)	-
T3	8 (10.13%)	-
T4	18 (22.78%)	-
N stage		
N0	69 (87.34%)	-
N1	10 (12.66%)	-
M stage		
M0	63 (79.75%)	-
M1	16 (20.25%)	-
Survival status		
Dead	27 (34.18%)	-
Alive	52 (65.82%)	-

**FIGURE 1:**
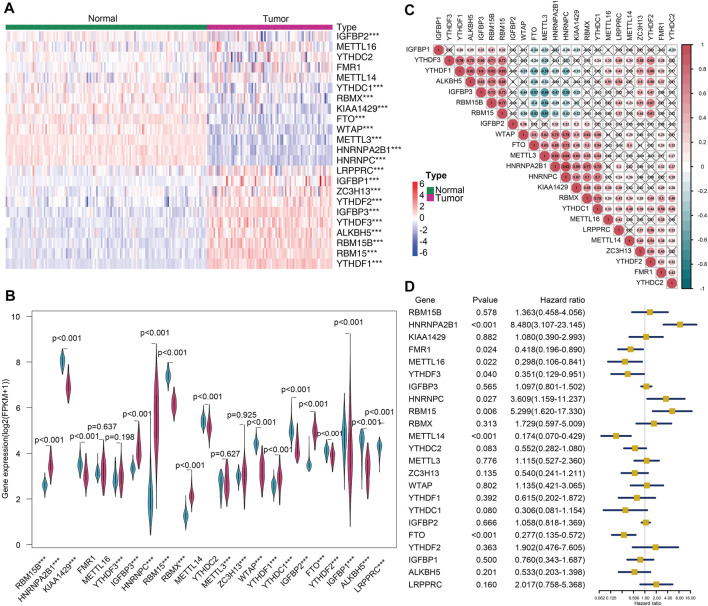
The landscape of m6A-related regulators of ACC. **(A)** Heatmap showing the differential expressions of m6A-related modulators between 79 tumors and 127 normal tissues (***p < 0.001). **(B)** Violin plot showing the differentially expressed regulators. **(C)** The correlation between the m6A modulatory genes in the cohort (×p> 0.05). **(D)** Forest plot depicting the prognostic capability of the 21 m6A-related modulators for ACC.

The heatmap (Figure 1A) and violin plots (Figure 1B) were employed for the purpose of visualizing the expression of various modulators of m6A RNA methylation. Relative to the normal adrenal tissues, the expression of most m6A genes demonstrated substantial changes in the tumor tissues. In general, the tumors had significantly high expressions of LRPPRC, IGFBP1, ZC3H13, YTHDF2, IGFBP3, YTHDF3, ALKBH5, RBM15B, RAM15, and YTHDF1 (*p* < 0.001). In contrast, the expressions of IFGBP2, FTO, YTHDC1, RBMX, KIAA1429, WTAP, METTL3, HNRNPA2B1, and HNRNPC were markedly reduced in tumor tissues. Nevertheless, no statistically significant differences were observed between the tumor and normal tissues in the remaining genes aforementioned. Furthermore, a strong correlation between various modulators of m6A RNA methylation, as shown in Figure 1C, indicated that these genes function together in a network. For example, the ALKBH5, IGFBP3, and RBM15/15B genes had a high likelihood of being up-modulated in the presence of enhanced YTHDF1 gene expression (Figure 1C). A univariate Cox regression analysis was utilized to determine the relationship between m6A modulators and the prognoses of ACC patients. The forest plot showed that only HNRNPA2B1, METL14, and FTO could be considered as protective factors (*p* < 0.001). In contrast, the remaining m6A RNA methylases had no substantial correlation with the prognoses of ACC patients (Figure 1D). These data indicated that although m6A RNA regulatory factors played an instrumental function in the occurrence and progression of ACC, modulators of m6A RNA methylation were not sufficient to predict the survival rates in ACC patients alone.

### Interactions Between N6-Methyladenosine Regulators and lncRNAs in ACC

The relationship between N6-methyladenosine regulators and lncRNAs was an intricate one. On the one hand, several studies illustrate that they perform a critical function in the expression regulation of N6-methyladenosine regulators. LncRNAs can interplay with proteins, RNA, DNA, and other molecules to regulate the structure and function of chromosomes, or as cis or trans-regulatory elements to regulate gene transcription, thereby affecting mRNA splicing, stability, and translation (Matsui and Corey, 2017; Ransohoff et al., 2018; Chi et al., 2019). On the other hand, N6-methyladenosine regulators regulate the RNA metabolism of lncRNAs, including the processes of RNA splicing and RNA stability, thereby resulting in the dysregulation of lncRNAs(Zhao et al., 2017; Chen et al., 2019). In view of this, the present research intended to better comprehend the function of m6A-related long noncoding RNAs (lncRNAs) in the incidence and progression of ACC.

**FIGURE 2:**
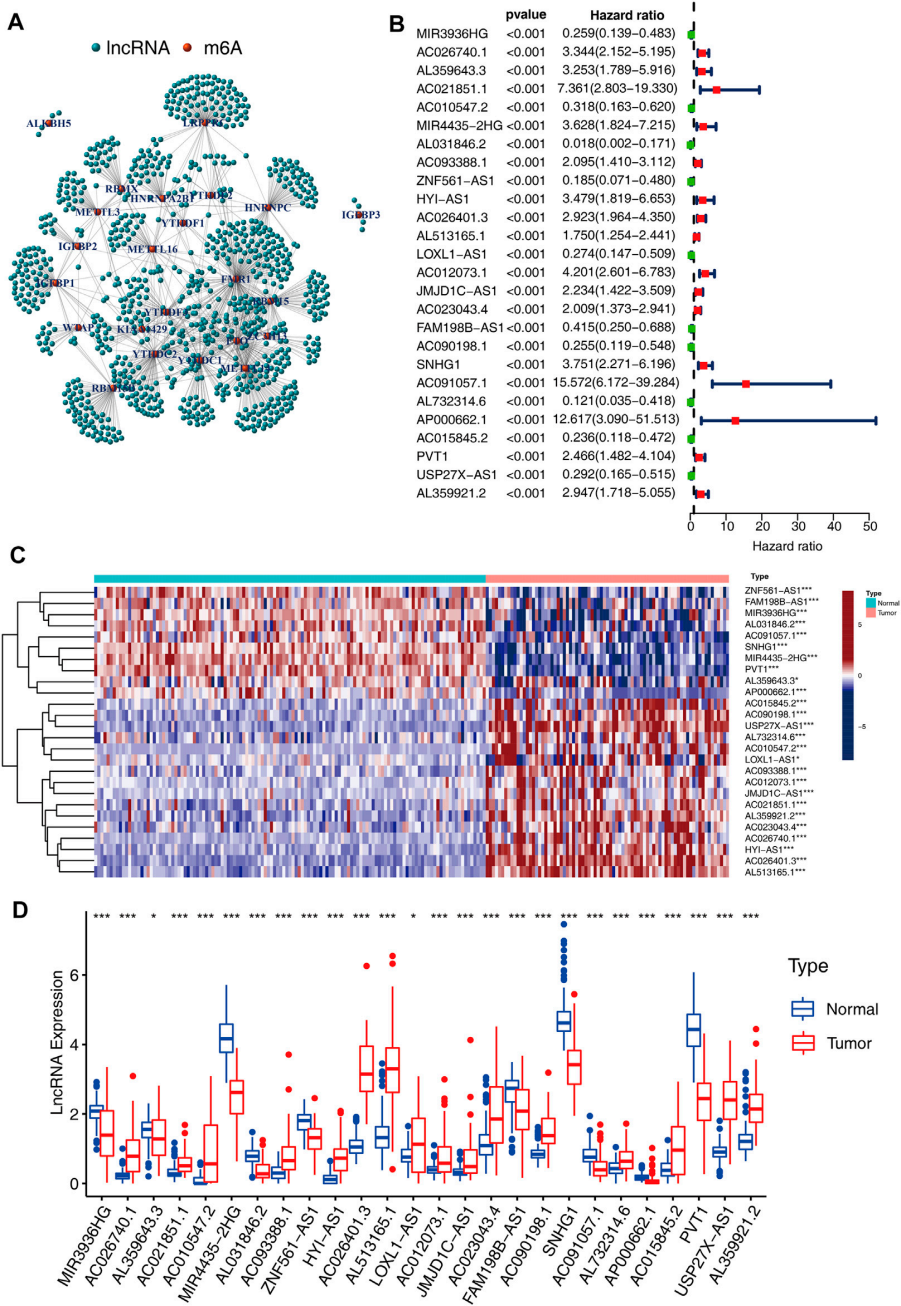
Interactions between m6A-related modulators and lncRNAs. **(A)** The complex modulatory network of 21 m6A-related regulators and lncRNAs. **(B)** 26 m6A-related lncRNAs have a significant correlation with the ACC patients’ OS. **(C)** Heatmap illustrating the differential expression of the 26 lncRNAs between 127 normal tissues and 79 tumor tissues (*p < 0.05; ***p < 0.001). **(D)** Boxplot depicting the differential expressions of the 26 lncRNAs.

The lncRNA annotated file for GRCh38 was obtained from the GENCODE webpage (https://www.gencodegenes.org). In the TCGA dataset, we identified 14,247 long noncoding RNAs (lncRNAs). Pearson correlation analysis was conducted and a significant correlation was found between the m6A-related lncRNAs and individual m6A-related regulators (*p*-value < 0.001 and correlation coefficient R > 0.5) (Figure 2A). A sum of 165 m6A-related lncRNAs was identified. Moreover, a univariate Cox regression analysis, as well as prognostic data, were used to screen for significant m6A-related prognostic lncRNAs among the 165 m6A-related lncRNAs. Eventually, a sum of 26 m6A-related lncRNAs was discovered to exhibit a significant correlation with the ACC patients’ OS (*p* < 0.001, Figure 2B), including 10 lncRNAs with protective effects and 16 posing a risk. The correlation coefficients between the 26 lncRNAs and m6A regulators as shown in Table 2. The heatmap (Figure 2C), as well as the boxplot (Figure 2D), were drawn to visualize the expression levels of these 26 m6A-related lncRNAs. Pearson correlation analysis was conducted for the corresponding 26 lncRNAs (Supplementary Figure S1).

**TABLE 2:** The correlation coefficients between the 26 lncRNAs and m6A regulators.

lncRNA	m6A Regulators	Correlation	*p*-value
AC010547.2	METTL14	0.504,832	2.09e-06
AC012073.1	RBM15	0.533,112	4.23e-07
AC015845.2	FMR1	0.539,199	2.95e-07
AC021851.1	METTL14	-0.5219	8.12e-07
AC023043.4	FMR1	-0.51318	1.33e-06
AC026401.3	RBM15	0.539,161	2.95e-07
AC026401.3	METTL14	-0.57383	3.23e-08
AC026740.1	RBM15	0.520,597	8.74e-07
AC026740.1	FTO	-0.55898	8.60e-08
AC090198.1	KIAA1429	0.519,391	9.36e-07
AC090198.1	FMR1	0.561,945	7.10e-08
AC090198.1	YTHDF3	0.607,224	2.98e-09
AC090198.1	FTO	0.60298	4.09e-09
AC091057.1	RBM15	0.550,114	1.51e-07
AC093388.1	FMR1	-0.50384	2.21e-06
AL031846.2	FMR1	0.504,047	2.18e-06
AL359643.3	FMR1	-0.50583	1.98e-06
AL359921.2	METTL14	-0.55098	1.43e-07
AL513165.1	FTO	-0.50393	2.20e-06
AL732314.6	METTL14	0.645,233	1.37e-10
AP000662.1	YTHDC2	-0.52498	6.80e-07
FAM198B-AS1	METTL14	0.588,506	1.17e-08
HYI-AS1	FTO	-0.51184	1.43e-06
JMJD1C-AS1	RBM15	0.561,825	7.16e-08
LOXL1-AS1	METTL14	0.522,835	7.70e-07
MIR3936HG	FMR1	0.530,958	4.81e-07
MIR3936HG	RBM15	-0.57874	2.31e-08
MIR4435-2HG	FMR1	-0.50438	2.14e-06
PVT1	RBM15	0.51502	1.20e-06
SNHG1	METTL14	-0.53757	3.25e-07
USP27X-AS1	METTL14	0.624,355	7.83e-10
ZNF561-AS1	YTHDF2	-0.51325	1.32e-06

### Consensus Clustering of the 79 ACC Patients

**FIGURE 3:**
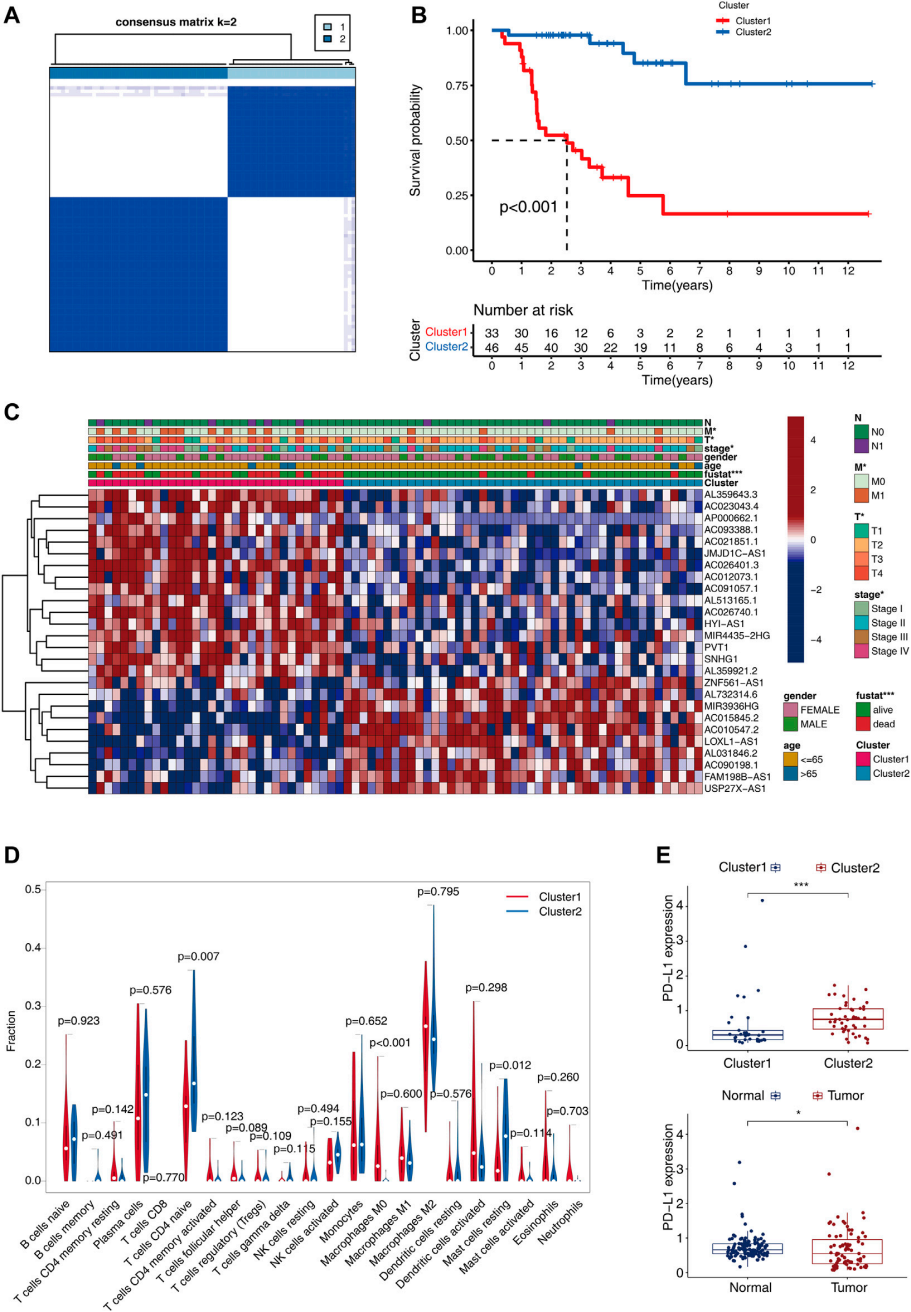
Consensus clustering for the 79 ACC patients premised on the 26 m6A-related lncRNAs with prognostic significance. **(A)** The consensus clustering matrix at k = 2. **(B)** The differences in OS between the two clusters. **(C)** Heatmap shows the correlation between the expressions of the 26 m6A-related lncRNAs and clinical-pathological characteristics of ACC. **(D)** With the aid of the CIBERSORT algorithm, substantial differences in the infiltrating levels of 22 distinct immune cell types between the 2 clusters are analyzed. **(E)** Differential expression of PD-L1 between the 2clusters (upper), and normal and tumor tissues (below).

To gain a better insight into the functions performed by m6A-related lncRNAs in the occurrence and progression of ACC, the ACC patients were classified into diverse subgroups by consistent clustering on the basis of their expression levels of 26 m6A-related lncRNAs with prognostic significance. CMplots were employed to visualize the matrix heatmap at k = 2, which showed obvious differences between the 2 clusters (Figure 3A). We evaluated the same results when the range of k was between 2 and 9. When k = 2, the empirical cumulative distribution reached the maximum approximation, thereby indicating maximum stability. Therefore, we divided the patients with ACC into two subgroups. We performed a survival test to analyze the clinical and pathological features of patients belonging to the 2 clusters. Interestingly, the survival status of cluster 1 was unfavorable, and the 5-year survival probability was about 35%, whereas that in cluster 2 was approximately 95% (Figure 3B, *p* < 0.001). For the purpose of exploring whether there was an association between different subgroups and clinical characteristics, the heatmap was plotted, which showed significant differences in the TNM stage, clinical stage, and survival status between different subgroups (Figure 3C).

We next investigated the differences in immune functions. With the aid CIBERSORT algorithm, we compared the 22 distinct immune cell types observed in different clusters.

The findings showed that B cells naïve, macrophages M2, T cell CD4 naïve, plasma cells, B cells, CD8 T cells, and mast cells resting accounted for a large proportion of the infiltrated immune cell (Figure 3D). Cluster1 showed an increased count of macrophages M0, while the number of mast cell resting and T cells CD4 naïve reduced as opposed to cluster2 (Figure 3D), which indicated the immune-deficient status in cluster1. Meanwhile, as compared with cluster2 and normal tissues, the gene expression of PDL1 was lower in cluster1 and the tumor tissues (*p* < 0.05, Figure 3E).

### Establishment of m6A-Related lncRNAs Prognostic Signature in ACC Patients

**FIGURE 4:**
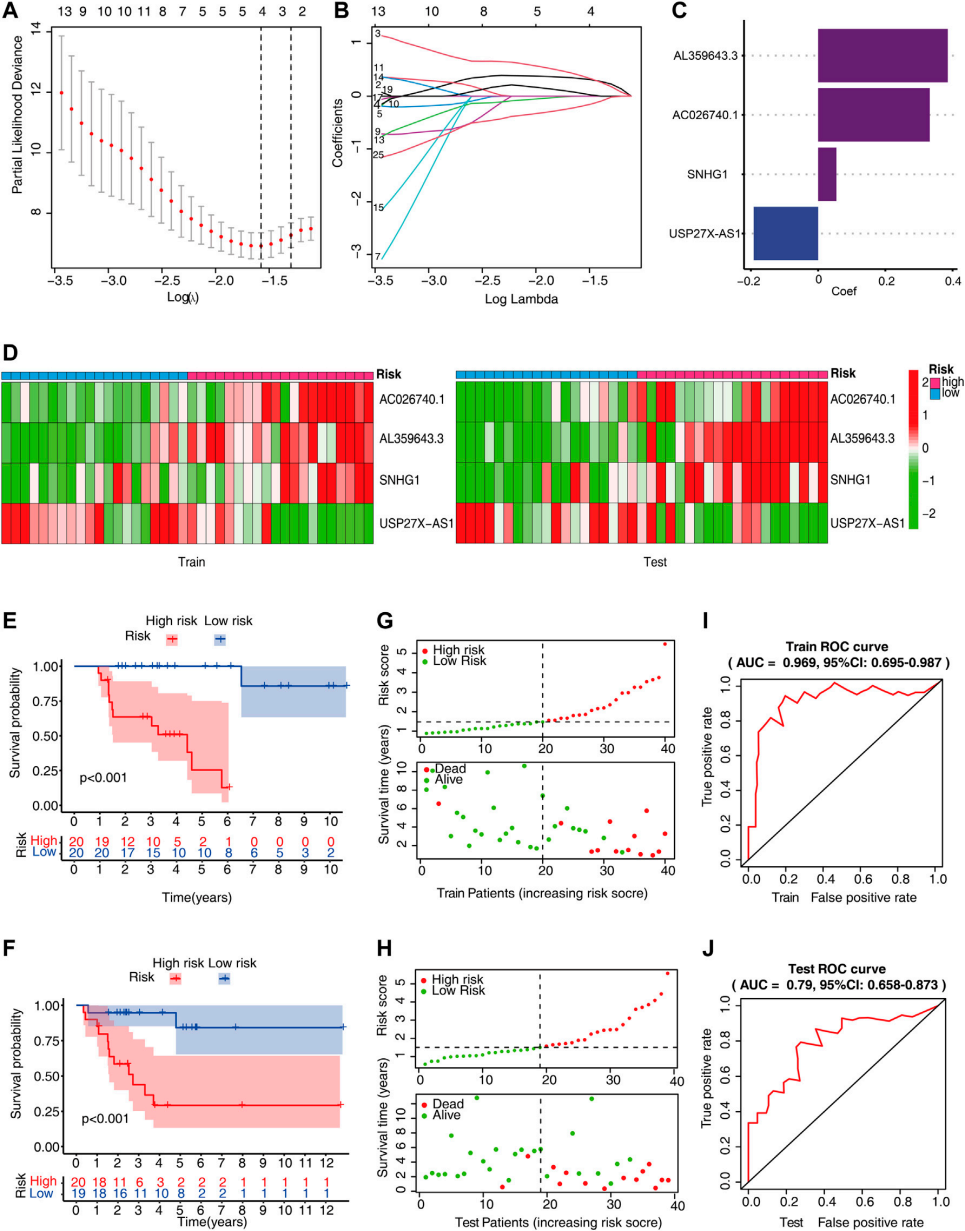
The risk score of m6A-related lncRNA. **(A,B)** Least absolute shrinkage and selection operator (LASSO) regression model. **(C)** With the minimal lambda criteria, the coefficients of the LASSO-Cox model are listed. **(D)** Heatmap shows the expression levels of four lncRNAs in the training and test sets. **(E,F)** Kaplan–Meier curves show that the high-risk cohort has unfavorable OS compared to the low-risk cohort in both training and test sets. **(G,H)** Survival status and risk score distribution of ACC patients in TCGA dataset. **(I,J)** The m6A-LPS receiver operating characteristic (ROC) curves were used in the prediction of the patient’s OS.

To construct the m6A-LPS for predicting the patients’ OS, we divided all the ACC patients into the training (*n* = 40) and test sets (*n* = 39). Subsequently, we carried out LASSO-Cox regression analysis on the substantial m6A-related prognostic lncRNAs that were identified for the purpose of computing the risk score for predicting the OS using the training set. Using the minimum lambda criteria, four m6A-related lncRNAs were chosen to construct the model (Figures 4A,B). Figure 4C depicts the coefficients for each of the lncRNAs studied. Heatmaps were drawn to visualize the expression level of each lncRNA in the two sets (Figure 4D). On the basis of the coefficients for each lncRNA, each ACC patient’s risk score was determined. According to the median risk score value, patients belonging to the TCGA cohort were classified into two subgroups: high-risk group and low-risk group. Whether in the training or the test set, the survival analysis indicated that ACC patients having elevated risk scores exhibited unfavorable clinical outcomes (a shorter OS time and reduced OS rates) (Figures 4E,F). Risk score and Survival status distributions were also plotted as demonstrated in Figures 4E,F. In addition, the ROC curves illustrated that m6A-LPS exhibited a powerful capacity for anticipating OS in both sets (AUC of training set = 0.969, Figure 4G; AUC of test set = 0.79, Figure 4H).

### Clinical and Pathological Features and Molecular Subtypes Linked to m6A-LPS

**FIGURE 5:**
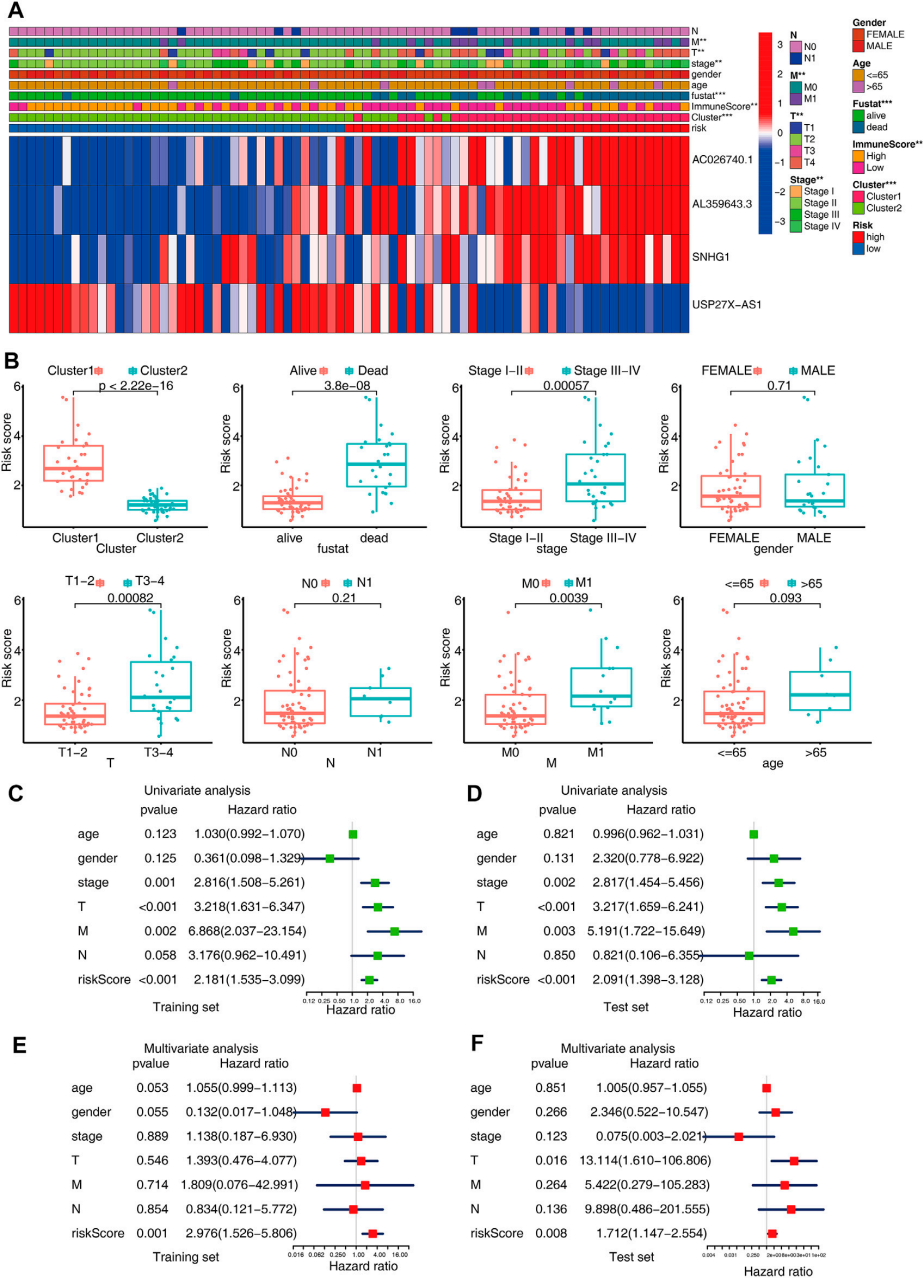
**(A)** Heatmap shows the relationship between the expression levels of the four selected m6A-related lncRNAs and clinical-pathological characteristics in ACC. **(B)** Boxplots showing the distinct risk scores between patients having various clinical-pathological characteristics (such as cluster, stage, gender, and TNM categories). **(C,D)** Univariate analysis shows that risk independently serves as a prognostic indicator in both training and test sets. **(E,F)** Multivariate analysis reveals that the risk score independently serves as a prognostic indicator in both the training and test sets.

First, to check whether there was a correlation between the different clinical features and risk scores, the heatmap was plotted, which showed that there were substantial differences in clinical stage, metastasis, cluster, immune score, and survival status among the ACC patients with high and low-risk scores (Figure 5A). The patients with advanced pathological stages, subtype with poor prognoses (cluster1), or high histological grades had a greater likelihood of exhibiting an elevated score (Figure 5B). Moreover, the risk score was independent of gender, which also showed that this prediction model could be applied to male and female patients. Next, we performed multivariate and univariate Cox analyses to examine if the m6A-LPS independently can serve as a prognostic marker for ACC patients in the training and test sets. The findings from the univariate Cox analysis illustrated that m6A-LPS was remarkably correlated with OS in both sets [hazard ratio (HR) in training set: 2.181,95% CI: 1.535–3.099, *p* < 0.001, Figure 5C; HR in test set: 2.091,95% CI: 1.398–3.128, *p* < 0.001, Figure 5D] and a multivariate Cox analysis also confirmed that m6A-LPS independently served as a prognosticator of OS (HR in training set: 2.976, 95% CI: 1.526–5.806, *p* = 0.001, Figure 5E; HR in test set: 1.712, 95% CI: 1.147–2.554, *p* = 0.008, Figure 5F). The above findings verified that the proposed m6A-LPS independently served as a predictor of OS for ACC patients, and may be useful for the evaluation of patient prognosis in clinical settings.

### Gene Set Enrichment Analysis and Immune Status in Patients With High- and Low-Risk Scores

**FIGURE 6:**
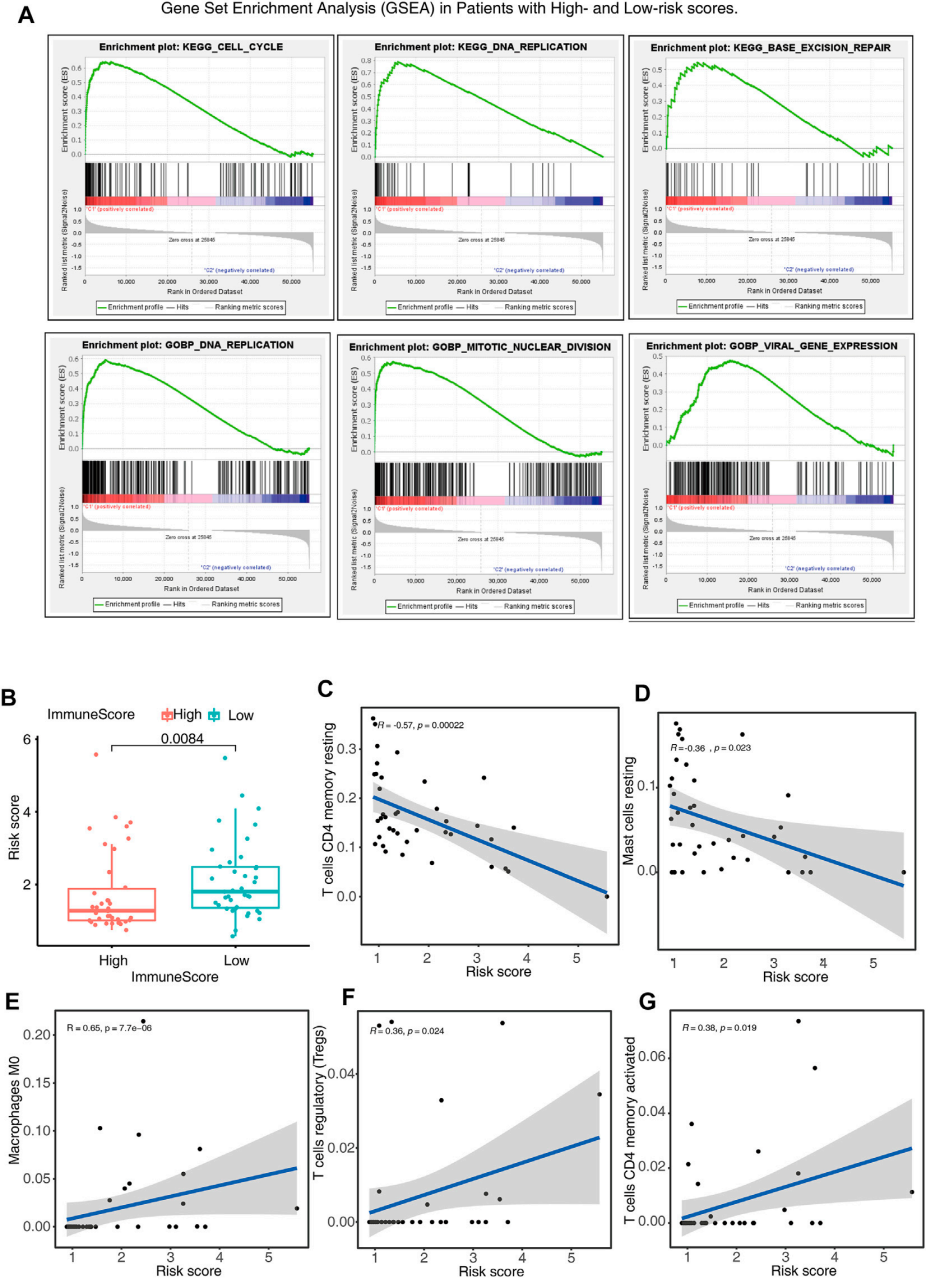
Gene Set Enrichment Analysis (GSEA) and immune status in patients belonging to the high- and low-risk score cohorts. **(A)** GSEA indicates the enrichment of tumor hallmarks in the high-risk cohort. **(B)** Boxplots show the differences in risk scores between the patients having low- and high-immune scores. **(C–G)** The relationship between risk score and partial immune cell types in low- and high-risk cohorts.

The GSEA was employed for the purpose of examining the possible biochemical mechanisms and pathways that may be implicated in the molecular heterogeneity seen between patients with ACC who were at low and high risk. The findings depicted that various tumor hallmarks were more prevalent in the high-risk cohort, including cell cycle, base excision repair, and viral gene expression pathways (Figure 6A). In addition, we also investigated the immune status and the correlation between immune cells and risk scores of low- and high-risk patients. The high-risk patients were found to have lower immune scores, reduced number of T cells CD4 memory resting and mass cell resting (Figures 6B–D), and a higher number of T cells CD4 activated, T cells regulatory (Tregs), and macrophages M0 (Figures 6E–G) compared to those in the low-risk cohort. The above findings might have implications in the cell biological and immune effects exerted by m6A-LPS.

### Functional Validation of m6A-Related lncRNAs

**FIGURE 7:**
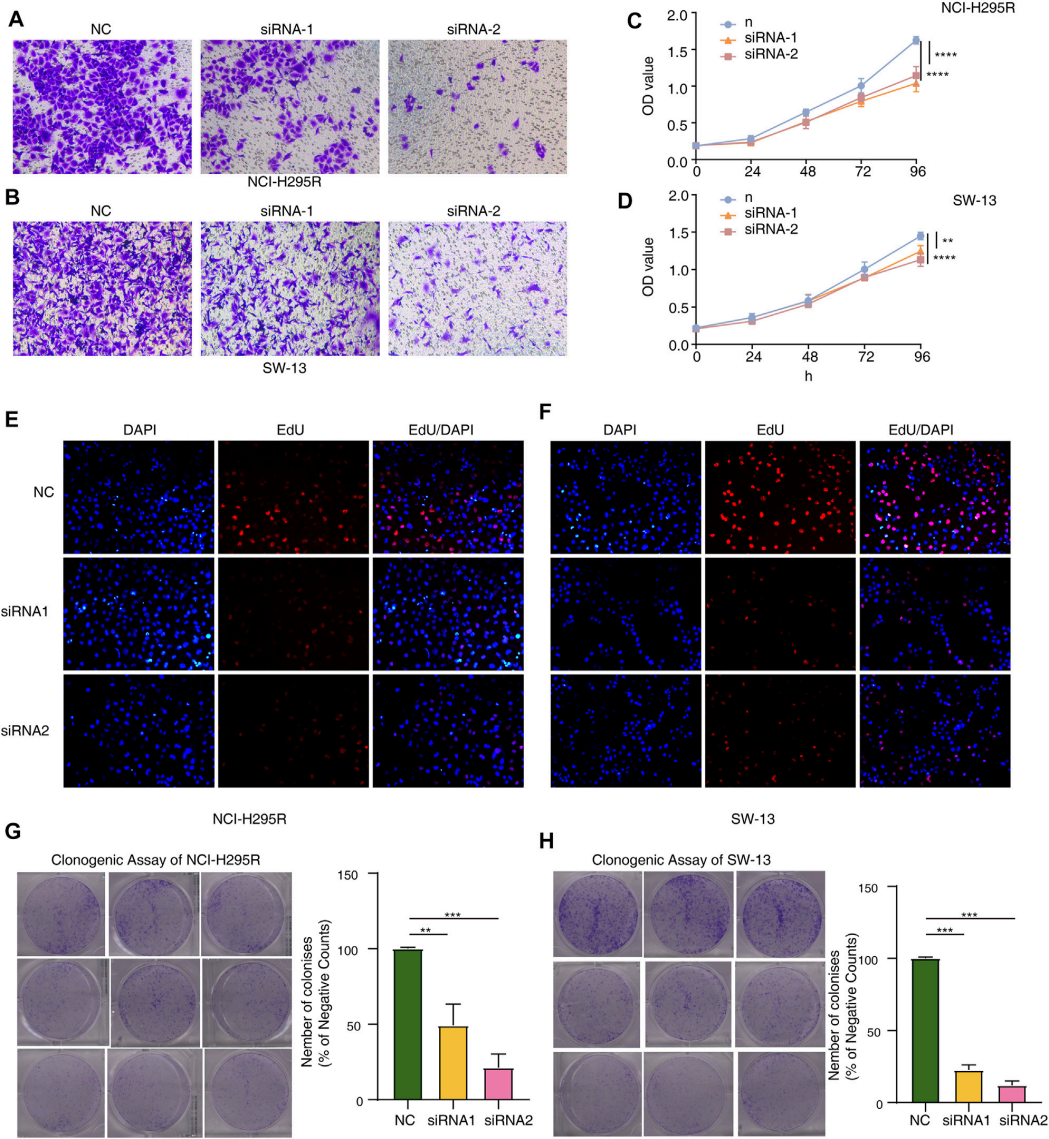
Functional validation with AL359643.3 knock-down. **(A,B)** Transwell assay was performed to measure the impact of AL359643.3 knock-down on the invasiveness of NCI-H295 **(A)** and SW-13 **(B)** cells. **(C,D)** CCK8 assay was utilized to measure the impact of AL359643.3 knock-down on the proliferation of NCI-H295 **(A)** and SW-13 **(B)** cells. **(E,F)** EdU-positive cells are in the si-AL359643.3-treated group relative to their si-NC counterparts in NCI-H295 and SW-13 **(F)** cultures. **(G,H)** Colony formation assay was performed of different groups in NCI-H295 **(G)** and SW-13 **(H)** cells (**p < 0.01, ***p < 0.001, ****p < 0.0001).

**FIGURE 8:**
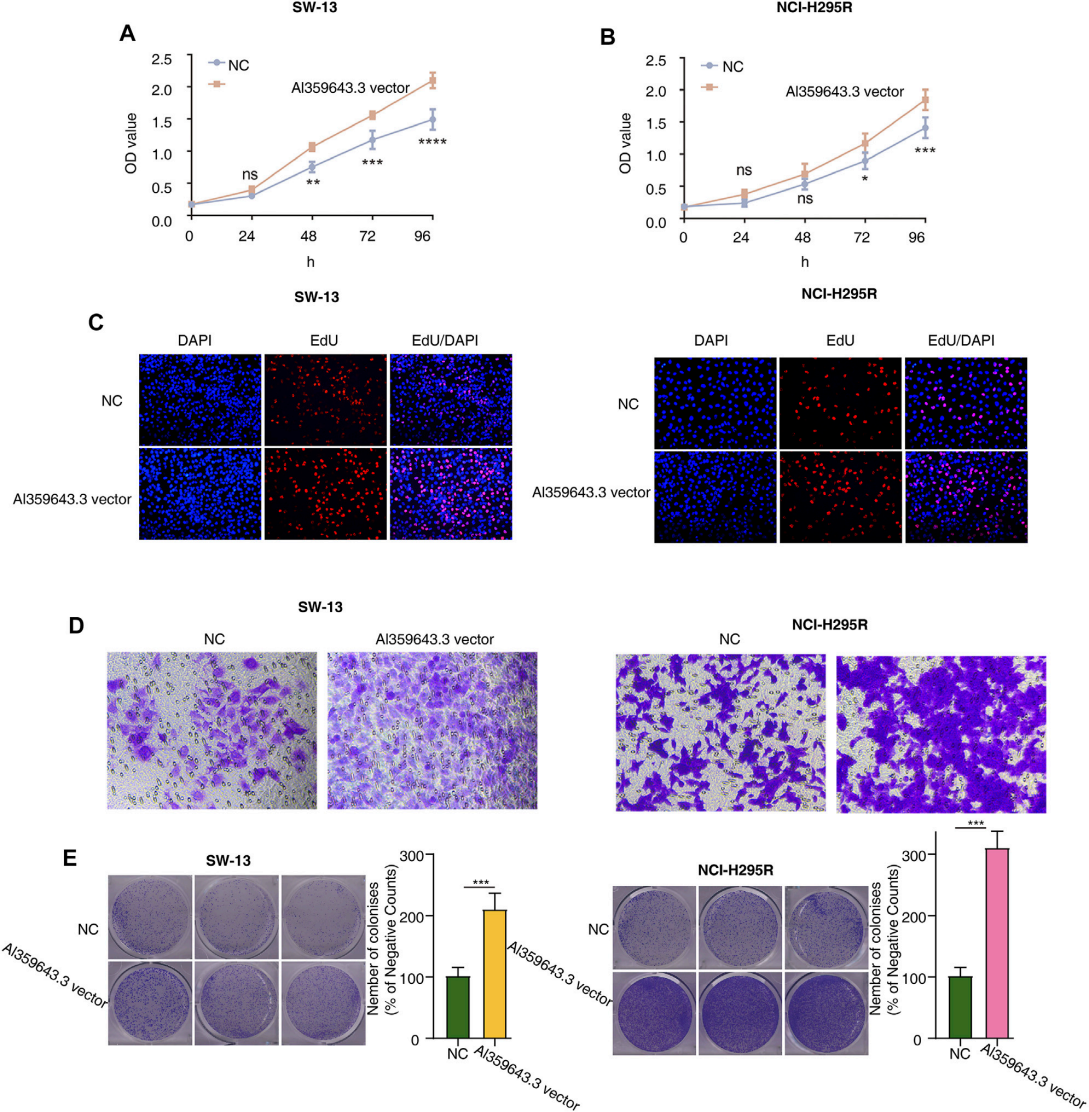
Functional validation with AL359643.3 overexpression. **(A,B)** CCK8 assay was utilized to measure the impact of AL359643.3 overexpression on the proliferation of SW-13 **(A)** and NCI-H295 **(B)** cells. **(C)** EdU-positive cells are in the AL359643.3 vector group relative to their Blank vector counterparts in SW-13 (left) and NCI-H295 (right) cultures. **(D)**. Transwell assay was performed to measure the impact of AL359643.3 overexpression on the invasiveness of SW-13 (left) and NCI-H295 (right) cells. **(E)**. Colony formation assay was performed of different groups in SW-13 (left) cells and NCI-H295 (right) (*p < 0.05, **p < 0.01, ***p < 0.001, ****p < 0.0001).

**FIGURE 9:**
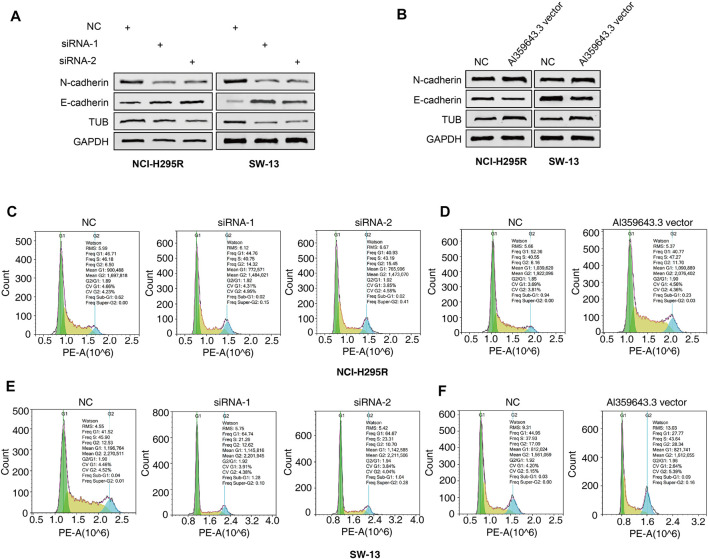
Effects of AL359643.3 on cell cycle and the expression of EMT-associated proteins. **(A,B)** Western blots of EMT-associated proteins with AL359643.3 knock-down **(A)** or overexpression **(B)** in both NCI-H295 and SW-13 cells. **(C,D)** Cell cycle progression detection with AL359643.3 knock-down **(C)** or overexpression **(D)** in NCI-H295 cells. **(E,F)** Cell cycle progression detection with AL359643.3 knock-down **(E)** or overexpression **(F)** in SW-13 cells.

To thoroughly determine the roles of the m6A-related lncRNAs, Al359643.3, which had the highest contribution in the model (Coef = 0.4), was selected to perform subsequent experiments. First, we designed the two si-RNAs constructs to knock down the expression of Al359643.3, which were transfected into NCI-H295R and SW-13 cell lines. Transwell assays showed that Al359643.3 might suppress the migratory and invasive abilities of tumor cells (Figures 7A,B). Moreover, the results of the CCK8 assay indicated that inhibition of lnc-Al359643.3 could markedly repress cell proliferation (Figure 7C). The EdU studies demonstrated a substantial reduction in the EdU-positive cells of the si-AL359643.3-treated group relative to the si-NC transfected cells (Figures 7D,E). In addition, the si-AL359643.3-treated group significantly potentiated the inhibition of colony formation when compared with the negative control group in both NCI-H295R and SW-13 cell lines (Figures 7G,H). Meanwhile, the overexpression experiments were also performed to verify the regulatory effect of Al359643.3 on cell proliferation and invasion (Figure 8), the results confirmed that Al359643.3 could promote the proliferation and invasion of tumor cells. As we know, EMT is one of the most common mechanisms closely associated with tumor invasion. So Western blot was performed to verify changes in the expression of EMT-associated proteins (including N-cadherin/E-cadherin/TUB) in cells with Al359643.3 knockdown and overexpression (Figures 9A,B), The results showed that Al359643.3 could change the expression of EMT-associated proteins. Moreover, considering the enrichment of cell cycle-related pathways in ACC patients with high m6A-LPS identified by GSEA, flow cytometry was performed to estimate the percentages of a cell population in the different phases of the cell cycle. The results suggest that Al359643.3 promotes the proliferation of tumor cells by prolonging the S phase of cells (Figures 9C–F). The above findings confirmed that the m6A-LPS may be of great significance for designing future treatment strategies for ACC.

## Discussion

N6-methyladenosine (m6A) is a common RNA modification of mRNAs and non-coding RNAs(Alarcon et al., 2015; Dai et al., 2018). These mRNAs regulate the stability translation, and splicing of protein-coding RNAs, and epigenetic impacts of some non-coding RNAs(Wang et al., 2014; Liu et al., 2020). Nevertheless, the functional impacts of m6A-related lncRNAs in ACC are yet to be realized completely.

In the present research, a total of 206 samples including 127 normal and 79 tumor samples were applied to examine the prognostic value of m6A-related lncRNAs in ACC. According to findings from the present research, the presence of prognostic significance for a sum of 26 m6A-related lncRNAs was validated in this cohort. According to the expressions of these m6A-related lncRNAs, the ACC patients could be stratified into two subtypes. 42% of samples belonged to subgroup-1, each having a worse prognosis and immunodeficient status. When the clinical features of the subgroups were compared, it was discovered that subgroup-1 exhibited a shorter OS time and a greater percentage of patients who were in the late stages of the disease as opposed to subgroup 2.

Furthermore, four of the 26 m6A-related lncRNAs were chosen to create an m6A-LPS for forecasting the ACC patients’ OS. The patients suffering from ACC were classified into two cohorts according to their median risk scores: low-risk cohort and high-risk cohort. The high-risk cohort patients exhibited poorer clinical outcomes and enriched tumor hallmarks, along with the upregulation of certain malignancy-related pathways. The results of a multivariate Cox regression analysis revealed that m6A-LPS independently served as a risk factor for OS. In addition, some functional experiments for one of the 4 m6A-related lncRNAs, Al359643.3, were performed to verify the accuracy and significance of this risk model. Several research reports have shown that m6A alteration might perform a modulatory function in the pathogenic mechanism of cancer (Wang et al., 2014; Wang et al., 2020), but its underlying lncRNA-dependent mechanism in ACC remains largely unclear. M6A regulators are implicated in the progression of several tumors through the modification of certain lncRNAs, for instance, KIAA1429 enhanced the progression of liver cancer by m6A modification of the lncRNA GATA3 (Lan et al., 2019). Thus, more attention should be paid in the future to the interconnections and roles of long noncoding RNAs (lncRNAs) and m6A alterations for the purpose of finding prospective cancer prognostic indicators or treatment targets.

However, there are some limitations to the present research. ACC should be studied in larger, and more diverse groups of patients are needed to verify the prognostic power of m6A-related lncRNAs. Moreover, to verify the functions of the lncRNAs, including the underlying molecular mechanisms, functional *in vivo* and *in vitro* trials, should be conducted.

In conclusion, the present research marked the first time that a thorough characterization and systematic investigation of m6A-related lncRNAs in the ACC has been performed. We discovered m6A-related long non-coding RNAs (lncRNAs) that had prognostic significance and developed a viable risk model with excellent prediction accuracy for prognoses and survival statuses. In the present research, the risk score was found to be strongly correlated with the clinical and pathological characteristics of ACC. Thus, it has the promise to function as a new and innovative biomarker in the near future. Our findings also offered critical evidence for additional research into the function of m6A-related lncRNAs in ACC, which may yield novel insights and lead to the development of effective tailored therapy options for ACC patients.

## Data Availability

The datasets presented in this study can be found in online repositories. The names of the repository/repositories and accession number(s) can be found in the article/Supplementary Material.
