# Supplementary figures and images for: Integrated Analyses Reveal Potential Functional N6-Methyladenosine-Related Long Noncoding RNAs in Adrenocortical Adenocarcinoma
Source: Front Cell Dev Biol. 2022 May 20;10:851748. doi: 10.3389/fcell.2022.851748 (PMC9163712; doi:10.3389/fcell.2022.851748)

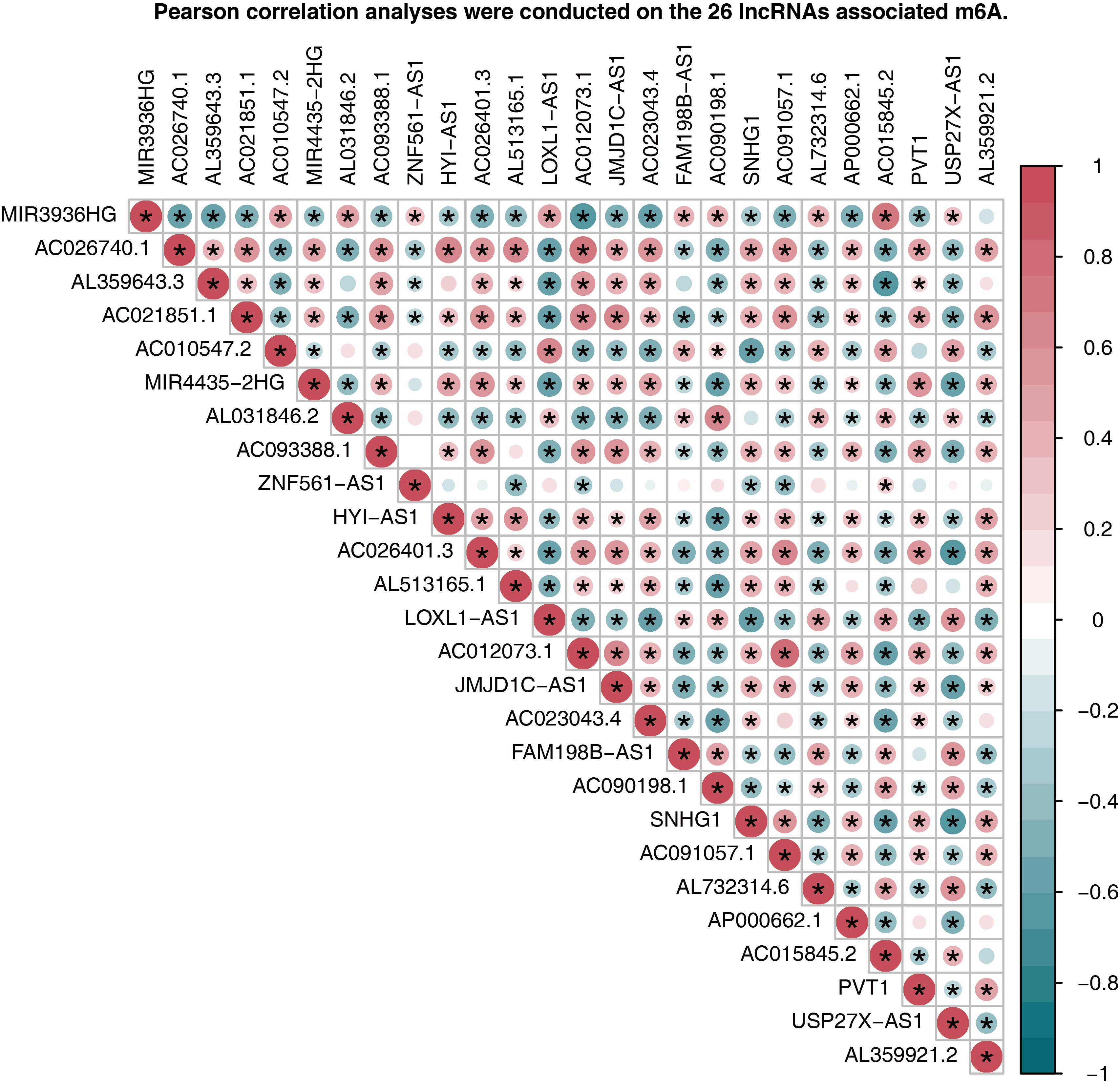

Supplement: Supplementary file 1 [file Image1.JPEG]
